# Embodied social exclusion: habit memory and the reproduction of caste in Dalit autobiographies

**DOI:** 10.3389/fsoc.2026.1884348

**Published:** 2026-09-28

**Authors:** Dhanshree C, R. Preetha

**Affiliations:** Department of English, School of Social Sciences and Languages, Vellore Institute of Technology, Chennai, India

**Keywords:** autobiography, caste, Dalit, embodied memory, habit memory, hierarchy, incorporated practices, inequality

## Abstract

Casteism stratifies people in India into hierarchical ranks, imposing customs, rituals, and traditions in accordance with the division of labour. This systematic continuity of casteism preserves memory that is passed down from generation to generation, not merely by ideology but also embodied by mnemonic practices. Dalits are the most afflicted community by both ideological and habitual oppression of the upper caste. Beyond legal abolition of untouchability, it persists as a system of descent-based ethnic inequality. This study explores autobiographies of Omprakash Valmiki’s *Joothan* (1997) and Urmila Pawar’s *The Weave of My Life* (2008), two Marathi Dalit writers who have penned their experiences of suffering and humiliation inflicted by upper caste. Using thematic narrative analysis through the lens of Paul Connerton’s concept of habit memory and incorporated practices, this study demonstrates that casteism is sustained in everyday habits by treating autobiographies as sociological evidence of ethnic caste discrimination. Embodied memory is performed through bodily acts, identifying five incorporated practices such as commensality, untouchability, labour division, spatial segregation, and postural discipline that function as bodily mnemonic systems through which hierarchical coercion is naturalised across generations. Existing scholarly works primarily focus on the abstract idea of casteism, identity quest, and trauma which leave a significant gap in understanding the role of embodied memory in sustaining inequality.

## 1 Introduction

Caste in India is a complex historical and socially stratified system with a hierarchical structure that determines the identity of social groups and has evolved from ancient spiritual archetypes into a rigid social order. Ambedkar (1936) defines caste as an endogamous unit, an “enclosed class.” He argues that while every society is composed of classes, a class becomes a caste when it creates a self-enclosed unit by superimposing the custom of endogamy over exogamy. Historically, the caste system originated from Chaturvarna, which divides society into four types: “(1) Brahmins or the priestly class; (2) the Kshatriya, or the military class; (3) the Vaishya, or the merchant class; and (4) the Shudra, or the artisan and menial class” (Ambedkar, 1917, p. 18). Over time, this relatively flexible varna system transformed into a hereditary caste order in which social status and occupation became determined by birth. Ambedkar (2014a,b) argues that, following the decline of Buddhism, *Manusmriti* consolidated Brahmanical authority by codifying hereditary duties and reinforcing caste hierarchy. For Ambedkar, this structure is not a simple ladder but a system of “graded inequality” (Ambedkar, 1917, p. 319) where each caste is motivated to maintain its status by feeling superior to the one below it, effectively paralysing any collective impulse for revolution. This structure fragments oppressed communities and weakens the possibility of collective resistance. Moreover, religious and ritual practices sustain the ideas of purity and pollution, thereby reinforcing caste hierarchy and social stratification through everyday practices such as food, touch, and bodily interaction.

The maintenance of the caste hierarchy relied upon a pervasive enforcement network of everyday discipline and systemic terror of punishment rather than religious sentiment alone. Drawing on Ambedkar, Rao (2009) explains that the “illegal laws of the Hin-dus” (p. 166) are enforced through practices by utilising the social boycott, where lawbreakers are sentenced to social death, which is ceasing completely from economic and social interaction. This disciplinary framework regulates the minute details of bodily conduct and movement. Historically, marginalised groups were required to carry brooms to erase their polluted footprints and wear spittoons to prevent their saliva from defiling the ground. Transgressions involving restrictions on food, touch, or marriage such as serving ghee or seeking access to common water sources triggered collective physical punishment and ritual murder, including beheadings and lynchings sanctioned by community elders. As Rao (2009) observes, these mechanisms functioned as a localised disciplinary tool to preserve a specific caste habitus, ensuring compliance with hereditary labour obligations by managing the social nausea of repulsion and the constant threat of total exclusion. Ultimately, such regulation ensured that the social order was reproduced daily through a phenomenology of fear and abject submission.

Ketkar (1909) defines caste as an enclosed social group which is determined by birth and maintained through strict hereditary endogamy, occupation, purity, and pollution. Similarly, Dumont argues that caste is fundamentally a matter of ideology, rooted in the religious values of purity and pollution that organise a “consensus of values” (Dumont, 1980, p. 20) known as *Homo Hierarchicus.* In this view, hierarchy is the fundamental social principle that encompasses all material power. Endogamous marriage perpetuates customs such as child marriage, widowhood, and Sati to prevent violations of the caste system. Initially, it was flexible, and an individual could change their varna if qualified. In the nineteenth century, the British Empire’s invasion of India further strengthened the caste system by expanding its bureaucratic governance.

Later in the 1950s, to abolish untouchability, the Indian Constitution introduced Articles 15 and 17 that address the discrimination through social and physical exclusion. The Indian government introduced affirmative action with the aim of providing opportunities to enhance the livelihoods of Scheduled Castes, Scheduled Tribes, and subsequently Other Backward Classes. Meanwhile, Casteism evolved as an electoral arena where political parties appeal as “‘back-ward’ on the basis of shared caste identity” (Bayly, 1999, p. 287). Constitutional reforms, Ambedkarite movements, and Dalit activities challenged caste hierarchy. In contemporary India, caste has undergone “substantialisation” (Bayly, 1999, p. 308), transforming from a relational system into a form of fixed ethnic identity or casteism that thrives within modern political and economic institutions. Extending these insights beyond the Indian context, Wilkerson (2020) conceptualises caste as an invisible social system that silently controls society on the basis of birth and ancestry, creating arbitrary boundaries. She states, “Caste is the bones, race the skin” (Wilkerson, 2020), suggesting that both are deeply embedded in society, although they differ in their overt expression. Wilkerson (2020) argues “caste systems live on in hearts and habits, institutions and infrastructures” (chapter 7) even after legal abolition, marginalised communities continue to live with residue of past codes. Moreover, the caste system continues to impede the social mobility as in rigid hierarchy, economic development as in a lack of opportunities, education as in illiteracy, gendered oppression, and political representation. Caste remains active in everyday life in matrimonial choices, domestic service, and even in the modern workplace, where social opportunities continue to be shaped by caste affiliation rather than ability and knowledge.

To understand why caste survives the “forced forgetting” (Connerton, 1989, p. 12) of modernity, we must shift our focus from abstract cognitive models to embodied practices. This transition from ideology to embodiment is theorised most effectively by Paul Connerton in *How Societies Remember* (1989). Connerton argues that social memory is the shared images of the past that legitimise a social order which is sustained primarily through ritual performances and bodily practices. He distinguishes between “inscribed practices” (Connerton, 1989, p. 72) such as texts, laws, monuments, and “incorporated practices” (Connerton, 1989, p. 73), which include postures, gestures, and habits. Connerton posits that every group entrusts its most essential values to “bodily automatisms” (Connerton, 1989, p. 102) and “habitual memory” (Connerton, 1989, p. 102). This type of memory is “sedimented in the body” (Connerton, 1989, p. 71) and does not require explicit reflection. It is knowledge embedded in the body understands through long-term habituation.

When applied to the caste system, Connerton’s framework reveals that stratification is maintained through a “choreography of authority” (Connerton, 1989, p. 74) expressed through the body. The everyday reproduction of caste stratification can be viewed in Dalit autobiographies in which the oppression is experienced physically, emotionally, psychologically, and through social violence. This study focuses specifically on its embodied dimensions which illuminate how caste hierarchy is reproduced through habitual bodily practices. The performance of certain physical movements is “legitimating performances” (Connerton, 1989, p. 35) that enforce hierarchical structures through bodily frameworks.

Connerton’s theory of incorporating bodily practices in Dalit life provides a deep understanding of social habit memory. Dalit literature emerged as a powerful literary movement in post-Independence India under the intellectual and political influence of Dr. B. R. Ambedkar. Rooted in lived experiences of caste oppression, it developed first in Marathi before expanding into Hindi and other Indian languages during the late twentieth century. The growth of Dalit literature was significantly accelerated by the “Mandal-Commission” (Tharu and Satyanarayana, 2011, p. 05) of the 1990s and the 1991 birth centenary of Dr. B. R. Ambedkar, which catalysed a national debate on caste and a rediscovery of Ambedkar as a foundational figure. “His revolutionary ideas stirred into action all the Dalits of Maharashtra and gave them a new self-respect. Dalit literature is nothing but the literary expression of this awareness” (Mukherjee, 2004, p. 1). Marathi became the pioneering language of this movement largely because Maharashtra was the epicentre of Ambedkar’s activities and the birthplace of the Dalit Panthers, where a militant organisation founded in Maharashtra in the 1970s that sought to bridge the gap between Dalit politics and radical class-based movements, whose work eventually expanded into Hindi through key political assertions and landmark translations, such as the 1991 Hindi release of Sharankumar Limbale’s “*Akkarmashi,* his autobiographical narrative of rural Dalit life.” (Mukherjee, 2004, p. viii). Autobiography became the dominant literary form because it allows authors to function as “speaking subjects” (Limbale, 2004, p. 8) rather than “objects of pity” (Limbale, 2004, p. 8), thereby providing social histories that infiltrate the Dalit voice into an official record that had previously ignored them. Foundational figures such as Omprakash Valmiki, Mohandas Naimishray in Hindi literature, and Baburao Bagul, Arjun Dangle, and Urmila Pawar in Marathi Dalit literature used their narratives to transform personal experiences of oppression into collective community histories. By chronicling the systemic humiliation of untouchability, they represent a shared “Dalitness” (Limbale, 2004, p. 11), a common condition of marginalisation effectively turning “private misery” (Tharu and Satyanarayana, 2011, p. 55) into critical social events that demand a fundamental reformulation of Indian democracy.

Omprakash Valmiki (1950–2013) was a prominent Hindi Dalit writer who challenged the stereotypes of casteism. Born into the churhra community (also spelled Chuhre), Dalit community in northern India historically associated with sanitation work and sweeping, Valmiki experienced direct oppression of the caste system. He gained significant recognition as a poet through collections such as *Sadiyon ka Santap* (1989) and *Bas Bahut ho Chuka* (1997), and as an essayist, he contributed vital narratives such as “Ek Dalit ki Atmakatha” and analysed the problems of Dalits in publications such as the *Navbharat Times.* His landmark autobiography, *Joothan* (1997), provides a visceral account of his journey towards self-awareness and has become a focal point for critical debate and social transformation. Valmiki’s extensive contribution to Hindi Dalit literature is defined by his role in laying its foundations and enriching it with short story collections and his critical study, *Dalit Sahitya ka Saundarya Shastra* (2001), which encouraged the community towards self-criticism and a revolutionary consciousness.

Similarly, Urmila Pawar (1945) is a pioneering Marathi Dalit feminist born into the Mahar community, the largest Dalit caste in the state of Maharashtra’s Konkan region, the community which encountered extreme social exclusion and assigned specific hereditary labour roles which included scavenging, cleaning cow pens, and disposing of dead animals. A dedicated activist, she founded a literary group called “Samvadini” (Sonalkor, 2008, Introduction) and has led efforts to organise Dalit women to address the intersecting issues of caste and patriarchy. Her celebrated autobiography, *Aaydan* (2003), translated as *The Weave of My Life*, uses the metaphor of bamboo weaving to document the everyday lives and systemic oppression of Dalit women. She also co-authored the seminal historical work *We Also Made History* (1989), which recovered the participation of Dalit women in the Ambedkarite movement. Her other literary work**s** include the short story collection *Sahava Bot* (*The Sixth Finger*), the travelogue *Mauritius: A Journey*, and “Shalya” and “Kavach.” These works have received prestigious literary awards, including the Maharashtra Foundation Award (USA) and the Priyadarshini Academy Award. Pawar’s contribution to Marathi Dalit feminism is defined by organising Dalit women in a specific stance which breaks the historical silence of Dalit women, ensuring their narratives “to understand the complex interweaving of caste and patriarchy and how it affects the lives not only of Dalit women but also of men and women of all castes living in contemporary India” (Sonalkor, 2008, Introduction).

The aim of the study is to examine caste stratification in the autobiographies of Valmiki’s, (1997) and Pawar’s *The Weave of My life* (2008), which exhibit the life histories of Dalit communities whose oppression was sustained through everyday habits using Connerton’s concept of embodied memory. Together, Valmiki and Pawar represent two major linguistic traditions of Dalit autobiography while foregrounding distinct gendered experiences of caste oppression. Their autobiographies therefore provide an appropriate corpus for examining how caste is embodied, remembered, and reproduced through everyday social practices.

## 2 Literature review

Previous studies have primarily examined the reproduction of casteism, which perpetuates social stratification and inequality in the Indian context (Dumont, 1980; Rege, 2006; Rao, 2009; Wilkerson, 2020). These studies focus mainly on ideological hierarchy, identity crisis, and the traumatic experiences of oppression (Dumont, 1980; Rege, 2006; Dutta, 2022; Vincent, 2025). These frameworks provide adequate insights into the systematic and psychological maintenance of the caste system, which are limited to abstract discourse and psychological states (Dumont, 1980; Rege, 2006; Dutta, 2022; Vincent, 2025). However, a review of these studies reveals a significant gap, namely, the lack of specific focus on the embodied memory of the caste system (Dumont, 1980; Rege, 2006; Rao, 2009; Dutta, 2022).

Historically, the ideological framework of casteism has been studied using Louis Dumont’s *Homo Hierarchicus,* which represents the peak “idealistic view” (Jaiswal, 2008, p. 5) of the caste. It portrays the binary system of casteism that is purity and pollution, which is viewed as “above all the system of ideas and values” (Jaiswal, 2008, p. 5). Moreover, it explains the construction of Brahmanical ideologies that uphold class exploitation, in which the social system is believed to be sacred as it is deeply ingrained and appears natural rather than forced.

Similarly, Basavaraja A. D. (2017) examines how ideology creates a “unique ideology of exclusivist doctrine” (p. 536), where high-caste norms are associated with morality and low-caste norms with immorality. This ideological reproduction is so pervasive that it operates as a social script followed by both teachers and peers in educational institutions. These approaches portray the human body as passive, simply complying with the ideology without objection, rather than as an active body.

Wilkerson (2020) compares Indian untouchability and American racial segregation and argues that both adopted similar social hierarchies to maintain rigid discrimination. Indian Dalits and African Americans are kept at the bottom of society through violence, fear, and legal systems. Another similarity that Wilkerson observes is that both groups were considered as polluted, where they were restricted to walk in public, accessing education, and using water fountains. The author also discussed that both groups were excluded from social equality and denied freedom. These studies explain caste primarily as an ideological and institutional system but provide limited attention to the body as a site through which caste is continually reproduced.

A few other studies focus on the politics of identity, particularly from a feminist perspective. Rege (2006) argues that Dalit women’s writings are often considered as testimonies, which are critical to comprehend how caste and gender go hand in hand as interconnected systems of hierarchies. Identity is seen as a “confrontational identity” (Rao, 2009, p. 1) used to refuse and reproduce terms of inclusion. Tanan (2025) cites *The Weave of My Life* (2008) by Pawar as a crucial Dalit feminist autobiography that employs personal narratives as a “tool of resistance and collective summary” (p. 90). These narratives reclaim agency and restore their social and political presence, which elevates the visibility to the emotional and experiential world of Dalit communities and allows readers to empathise their struggle and pain. Mohan (2014) articulates that these narratives “breathed a new life into the emotional world of Dalit community” (p. 76). However, the main focus of these narratives prioritises the inscribed record of memory, where the writing acts as political intervention and often overlooks the incorporated record of memory. These studies interpret Dalit autobiographies as testimonies of identity, resistance, and political assertion. However, they pay little attention to how bodily practices themselves preserve caste relations.

Furthermore, Rege (2006) implies that Dalit male autobiographies usually represent caste through the lens of the social life, focusing on public struggles, labour, public institutions, and political assertion. Their testimonios represent caste experiences that challenge dominant caste histories, resurrect Dalit triumphalism, and invoke guilt in the minds of the upper castes by recording ancestral wrongs. Within these male narratives, women are often only “selectively remembered as sacrificing wives and mothers or victims” (p. 75) rather than active political agents. Dalit female autobiographies expand this representation by focusing on the “interlocking axes of caste, class, and gender” (p. 67). She explains that women’s life writing introduces narrative concerns regarding domestic labour, the household, and the body (embodiment) that are often erased in male-centric accounts. For instance, women’s narratives detail the specific toil and menial tasks of lower-caste labour, such as cleaning animal pens, which places them in a unique position of social exclusion compared to both upper-caste women and Dalit men. Moreover, these testimonios explicitly critique Dalit patriarchies and the regulation of female sexuality as the gateway to the caste system, a concern rarely addressed in male writings. Women’s writing also portrays education as a means of questioning social misrecognition and overcoming caste and gender oppression. Comparing the autobiographies of Valmiki and Pawar is essential because it reveals that the real experience of Dalitness is not singular; instead, it shows how gender reinscribes memories of resistance and domestic humiliation, thereby challenging a monolithic understanding of the Dalit movement and reformulating the very notion of Indian democracy.

Additionally, trauma has become a prominent discussion among various studies in Dalit writings. Dutta (2022) examines Dalit autobiography through the interconnected themes of atrocity, storytelling, and trauma as crucial conditions of Dalits’ lives created by caste oppression, and physical and verbal abuse. It emphasises trauma as both psychological and social states which are expressed through the narrative of testimony, resistance, and protest where Dalits’ condition is in constant “atmosphere of fear” (p. 127). Similarly, Vincent (2025) argues that *Joothan* (1997) is a collection of “excruciating memories” (p. 264) that show the profound mental suffering based on caste humiliation and oppression. When the past experiences linger unhealed, the trauma remains unresolved through constant fear, pain, and discrimination. Similarly, Vyas (2020) discusses the prolonged emotional wounds and inner conflict due to psychological rupture of humiliation that are faced in Dalit writings. In *Joothan* (1997), trauma is expressed through cultural psychology, featuring identity struggles, transitions, and processes of redefining in Valmiki’s life. While trauma studies illuminate the psychological consequences of caste oppression, they do not sufficiently explain how repetitive bodily practices become mechanisms through which caste is reproduced in everyday life.

Phenomenological exploration of caste embodiment has also been discussed in anti-caste scholarship. Thirumal (2020) explains that caste is not just an idea but something lived through the body in everyday ways. He argues that caste consciousness is expressed through “arts of living” (Thirumal, 2020, p. 36) such as the choice of food, way of thinking, and social interactions, which become embodied dispositions shaping everyday life. By locating caste within the lived body, Thirumal demonstrates that caste hierarchy is reproduced through ordinary bodily practices rather than solely through explicit beliefs or institutional structures. While this perspective establishes the significance of embodiment in understanding caste, it primarily examines the lived experience of caste embodiment. Additionally, Jaaware (2019) defines caste as a “material regulation of the bodies” (p. 25) centred on the simple physical act of touching. He discusses the sensory boundary when people unconsciously shut off their senses to maintain caste distance. He frames untouchability as a “denial of bodily presence” (p. 155), a systematic refusal to acknowledge the physical traces of marginalised individuals that are left behind in a shared environment. These studies establish caste as a sensory orientation and a “regime of touch” (p. 58) that dictates physical proximity before those interactions are sedimented into habitual memory. Together, these studies establish caste as an embodied and sensory experience enacted through everyday bodily practices.

Moreover, several scholars have utilised Bourdieu’s habitus to examine “culturally specific practices of bodily comportment inculcated early in life” (Rao, 2009, p. 310). Habitus suggests that inequality is reproduced through the “reproduction of bodily practice, belief, and comportment” (Rao, 2009, p. 309). Anupama Rao acknowledges the significance of Bourdieu’s habitus in understanding how inherited prerogative is habitualised through the upper-caste habitus of secular modernity. For instance, the habitus of the secular Brahmin allows them to perform bureaucratic competence in public while vehemently propagating their rituals and customs in the familial space. Conversely, the Dalit habitus is often marked by ingrained humiliation and a “multi-layered and enduring sense of vulnerability” (Tanan, 2025, p. 91). However, Rao also notes a critical limit of Bourdieu’s habitus, which is “less helpful in explaining alterations in corporeal practice” (Rao, 2009, p. 310). While habitus explains how the body sits or fits into existing structures, it does not fully account for the way caste-specific embodied memory, the actual physical repetition of specific labour such as basket weaving or manual scavenging, functions as a generative force of social memory.

The comprehensive review of Olick and Robbins (1998) recognises Paul Connerton as a significant figure in the shift towards a “historical sociology of mnemonic practices” (p. 105). The authors interpret Connerton’s contribution as shifting attention from memory preserved in texts and monuments to memory incorporated through bodily practices. His work is categorised under “inertial persistence” (p. 129), where social memory is reproduced through the sheer force of habit and custom. Viola (2024) positions Connerton as the primary scholar who moved the study of cultural transmission from biological conceptions of memory to a sociological paradigm centred on gestures and habits. Viola interprets Connerton as a critic of “hermeneutical approaches” (p. 105) that privilege language, texts, symbols, and interpretation. Instead, Connerton is acknowledged for his focus on habit memory, the bodily capacity to reproduce performances through techniques, proprieties, and ceremonies of the body. The existing discussions of Connerton primarily examine embodied memory in relation to ritual, performance, and social practices, while little attention has been given to its application in Dalit autobiographies and caste embodiment. This gap provides the basis for the present study, which employs Connerton’s lens to explore how caste identities are enforced and resisted through the bodily habits and embodied social memory documented in Dalit life writing.

Therefore, the existing literature effectively explains caste inequality through ideology, identity, trauma, and embodiment. It also employs Bourdieu’s concept of habitus to explain how caste is ingrained through bodily dispositions. However, there remains a noticeable lack of attention to how repetitive bodily practices function as embodied memory in reproducing caste across generations. To observe beyond the ideological and psychological explanations of caste, the present study employs Connerton’s concept of incorporated memory to examine how the body itself becomes a site for the reproduction of caste. Both prominently exhibit incorporated practices reinforced through repetitive bodily performances, leaving enduring embodied scars that sustain inequality. By applying Connerton’s lens, this study examines how caste inequality is not merely an ideology but also an embodied social memory performed and reproduced through everyday bodily practices.

## 3 Theoretical intervention: embodied memory

Paul Connerton is a social anthropologist who treats memory as a cultural phenomenon that is transmitted through incorporated practices rather than inscribed memories. Most scholars treat cultural memory as inscribed practices present in texts, documents, archives, and written material. This tendency is challenged by Connerton, by arguing “…images of the past and recollected knowledge of the past are conveyed and sustained by ritual performances, and that performative memory is bodily” (Connerton, 1989). He claims images of past memory recollected are not just cognitive, it is “sedimented in the body” (Connerton, 1989, p. 72), which is preserved and sustained through ritual performances. To legitimate a present social order, the participants must presuppose a shared memory of the past to function.

Connerton posits that bodily social memory is essential, which is often neglected by scholars. His theory of social memory is built upon an intellectual synthesis of Marcel Mauss’s “techniques of the body” and Pierre Bourdieu’s “habitus.” Marcel Mauss initiated this by demonstrating that there is no truly natural way for a human to use their body; rather, the body is “man’s first and most natural technical object” (Mauss, 1935, p. 75). He observed that every gesture is not mere habit but technical skills imposed from above by the community to achieve social efficiency. Later, Bourdieu (1977) refined Mauss’s observations into a comprehensive theory of the habitus, which he defines as a system of “durable, transposable dispositions” (Chapter 2). Bourdieu argues that social structures become internalised through habitual bodily practices, appearing natural rather than consciously learned.

Connerton (1989) integrates these views to explain habit memory: the capacity to reproduce a performance without conscious reflection. He argues that when a society entrusts its values to these “bodily automatisms” (p. 102), it ensures that its history is kept in mind through a habitual memory that is ingrained into the physical conformation of its members. Memories can be carried by commemorative ceremonies since they are performative, where the past remembers through habits, gestures, postures, and rituals. Those habits are not just dispositions; rather, they are repetitive acts that follow unconsciously.

The choreography of authority is expressed through postures of the body which is evident in communal memory. Power and rank are often conveyed with certain postures, particularly to stand, sit, bow, and curtsy relative to others. The inflexions of upright and down posture may slightly vary but reminds respect, dominance, and submission of performers and observers of the society. Even without words, the authority is apparent with specific postures which become mnemonics of the body.

Connerton has distinguished two different types of social practice in which memory is sedimented or accumulated in the body: incorporated practices and inscribing practices. Incorporated practices are everyday gestures such as a smile, handshake, greetings, or rituals acting through the body. The transmission of embodied performance occurs intentionally or unintentionally, when the body exists. It is mostly traceless and no evidence left behind once the body ceases to perform. On the other hand, inscribing practices are objects or material where memories are stored outside the bodies as in texts, indexes, photographs, tapes, and other technological storages to “trap and hold information” (Connerton, 1989, p. 73), even when the body or individual is destroyed.

Connerton claims that social structure retains an ‘inertia’ that present orthodoxies cannot fully explain. This inertia is sustained through the “accumulative practice” (Connerton, 1989, p. 34). This accumulative practice can be applicable to caste-like systems where rituals and habits are re-enacted and repeated every day. It is not merely an idea of remembrance of the past; instead, the past event or rite is metaphysically present in contemporary society through embodied practices where the hierarchy remains unchanging and authentic in the minds of participants.

For both dominant and marginalised groups, the body becomes the “privileged site” (Connerton, 1989, p. 70) of memory. The deep inertia of hierarchical systems like caste is not a product of cognitive failure but of performative success. Connerton’s framework, supported by the insights, reveals that incorporating practices are the primary engines of social persistence.

### 3.1 Methodology

This study applies the thematic narrative analysis of autobiographical narratives considered as empirical data of case-centred research. It treats Omprakash Valmiki’s, (1997) and Urmila Pawar’s The Weave of My Life (2008) as sociological evidence which restores the voices of marginalised individuals. Documenting the real sufferings of casteism turns out as Riessman (2008) said, “truths about human experience” (Chapter 1) that exclusively focus on “what” is said or written. Analysing marginalised autobiographies contributes to social identity and community awareness which share similar experiences of the oppressed group. By using real names and real-life incidents, the narrative becomes a testimony and a record of Dalit experiences, challenging the deep-rooted refusal to acknowledge caste realities in Indian society.By using real names and real-life incidents, the narrative becomes a testimony and a record of Dalit experiences, challenging the deep-rooted refusal to acknowledge caste realities in Indian society. Similarly, Pawar (2008) indicates her autobiography as a “social document” to exhibit the struggle and participation in Dalit movements as a woman. These texts are not merely subjective narratives; rather, it acts as archives of resistance that interrogate the social structure of hierarchy. Using Connerton’s embodied memory, this thematic narrative analysis focuses on the intact story, which provides necessary incidents to comprehend the caste structure and identifies the incorporating practices of casteism which retains inequality through everyday habits.

## 4 The five pillars of embodied hierarchy

Caste functions as a social habit memory sedimented in the body through specific repetitive incorporated practices:

### 4.1 Commensality

Consumption is the incorporating act par excellence. Marcel Mauss (1935) observes that the ways in which individuals eat and drink are not natural but are “assembled for the individual... by all his education, by the whole society to which he belongs” (p. 76). In a caste context, the rules of commensality function as what Connerton (1989) calls “proprieties of the body” (p. 79). Ambedkar notes that restrictions on inter-dining are because of an enclosed caste structure which works in habitual ways of doing things according to the system. These rituals act as “rule-governed activity” (Connerton, 1989, p. 44) that draws attention to the purity or pollution of the participants. He gives the instance of an untouchable host who served ghee for other untouchable guests, which became aggressive and a violation of higher caste status in the view of other castes. Untouchables are brutally attacked and claimed as “impudent” and “foolish” (Ambedkar, 1936). Moreover, Brahmanical codes consider food from untouchables as “Semen or urine” (Ambedkar, 1936). This “absence of messing” (Ambedkar, 1917, p. 8) is not merely a rule but a natural result of the group’s exclusiveness.

### 4.2 Untouchability

The “idea of pollution” (Senart qtd in. Ambedkar, 1917, p. 7) is a sensorial ordering where certain bodies are marked as permanently impure to avail the higher caste privilege. Fundamentally, Casteism follows the idea of purity and pollution, in which Wilkerson (2020) asserts “the lowest-caste people were to remain a certain number of paces from any dominant-caste”. Some were forced to wear bells to warn of their presence or sweep off their footprints, so they will not pollute others. In the case of leatherworkers, the “stench of raw hides” (Kapoor, 2025, p. 339) becomes an “ontological wound” (Kapoor, 2025, p. 340), forcing the individual to hold testimony against their own self through their bodily state. The Hindu localities smell of “Jalebi and Kachori” (Kapoor, 2025, p. 339); meanwhile, untouchables encircled with “malodor” (Kapoor, 2025, p. 340).

### 4.3 Labour division

“Division of labourers” (Ambedkar, 1936) is one of the main causes of Casteism. It is defined as “watertight compartments” (Ambedkar, 1936). This system appoints tasks to individuals in advance based on their social status. Labourers are ranked in the hierarchical order based on the dignity of the work, in which few consider superior and others inferior. These repetitive actions of a specific labour are sedimented in the body as a habit memory that transmits unconsciously to subsequent generations. Techniques of manual labour, whether digging, weaving, or marching, are “physio-psycho-sociological assemblages” (Mauss, 1935, p. 85) that must be laboriously acquired through “prestigious imitation” (Mauss, 1935, p. 73) of those in authority. This incorporated hereditary skill cannot be replicated by other communities who rely on “bookish knowledge” (Kapoor, 2025, p. 349).

### 4.4 Spatial segregation

This spatial distancing is an incorporating practice that does not need to be verbalised to be understood; the “meaning-made-flesh” (Connerton, 1989, p. 104) of segregation precedes any ideological justification for it. Social memory is localised through mapping; we situate our recollections within the “material spaces that particular social groups occupy” (Connerton, 1989, p. 37). Ambedkar notices the geographical distinction where untouchables were historically forced into settlements on the outskirts of Hindu villages. These spaces are further marked by sensory effects, “the Untouchables, the Unseeables, the Unapproachables—whose presence, whose touch, whose very shadow is considered to be polluting by privileged-caste Hindus” (Roy, 2014, *The Doctor and the Saint*). Moreover, few communities were forced to tie brooms around their waist to sweep the place where they stood and to erase their footprints. However, these practices are historical; they are performative and ritualistic.

### 4.5 Postural discipline

Connerton notes that “power and rank are commonly expressed through certain postures relative to others” (Connerton, 1989, p. 73). Power and hierarchy are often expressed through the body that perpetuates the “choreography of authority” (Connerton, 1989, p. 74). Subordination is not just a message but is displayed through the “visible, present substance of one’s body” (Connerton, 1989, p. 59) such as kneeling or maintaining a submissive attitude. Even exhibiting a dignified attitude or posture could be penalised as defiance of the caste norms. Wilkerson (2020) states subordinate castes were often forbidden from looking the face or eyes of dominant caste individuals. Untouchables are expected to perform submissively.

## 5 The choreography of caste: bodily habits of Dalit subjugation

Connerton studies how societies remember their past through habit memory, in which the memory is sustained by “bodily automatisms” and preserved in the continuing ability to perform specific rituals and routines. In the lens of habit memory, the sedimentation of caste hierarchy in the physical body is visible through incorporated practices.

### 5.1 Food incorporation: the bodily memory of hierarchy

Connerton notes that consumption is a primary incorporating act where social rules determine social status by training the body. In Dalit life, the physical act of eating leftovers or specific lowly foods creates a habitual register of social rank. In Valmiki’s narrative, the titular act of joothan is ritually practised in the Chuhra community, who eat leftover food, the residues of higher communities. This destined act becomes mnemonic and symbolises the sustained degradation in the lives of people. Valmiki recalls how the elders narrated stories of the leftovers they received in “thrilled voices” (Valmiki, 1997, p. 10), effectively teaching the children to relish their own humiliation as a legitimate social currency. During events, Churas have to wait for the leftover food until the party members complete their meal. They linger with huge baskets to collect those leftovers and even sun-dry them, such as pooris, puffed bread, sweets, and bhatashas for future usage. The ritual of joothan continued throughout his lifetime, causing abiding humiliation. The practice of forcing Dalits to consume leftovers disciplines the body through food practices, repeatedly reinforcing notions of purity and pollution while naturalising social inferiority. This “habit of servitude” (Connerton, 1989, p. 74) is so deeply sedimented that, as an adult, Valmiki experiences a visceral, bodily reaction of nausea when remembering the food of the Tyagis. The authority of the Tyagi community in *Joothan* is enacted through a ritualised performance of spatial and temporal dominance during communal feasts, where Dalit bodies are relegated to the margins of the social event.

Correspondingly, Urmila Pawar dedicates an entire chapter to describe food and eating routine which exhibit poverty, malnutrition, and insecurity among and beyond her community. She explains her husband’s family as the “bite-counters” (Pawar, 2008, Chapter 1), where men in the family sit on their haunches to dine from one plate due to a lack of plates. Scarcity of food produced a form of bodily discipline carefully counting how many bites each person took. Any individual taking “one morsel more” (Pawar, 2008, Chapter 1) would spark a bitter fight, turning each meal into a repetitive ritual of surveillance and survival. This habitual behaviour assures the reality of their sparse and scarce existence is physically performed every day. She elaborates on the experience of eating meagre meals, including rice with red ratamba curry, a native fruit used as flavouring, where the body learns to “survive this curry” through a repetitive cycle of insufficiency. She recounts a story of a son-in-law who is served less rice with plenty of curry for a meal, distracting him while eating to hide the fact of scarcity by interrogating him about his brothers. This habitual relationship with food functions as a “material milieu” (Connerton, 1989, p. 37) that localises the group’s memory of poverty. Children are made to eat whatever is given without complaining to battle their hunger. Rich sweets are never available for them. Scarcity of food creates bodily uncertainty, which leads to a sedimented habit of immediate, uncritical consumption of food. Poverty is not just a social system but “physical realities” (Connerton, 1989, p.101) preserved in everyday life through these incorporating practices.

### 5.2 Labour incorporation: repetitive bodily conditioning

Connerton argues that habit is a form of knowledge and “remembrance in the hands” (Connerton, 1989, p. 93). In these autobiographies, caste-based labour functions as a repetitive bodily conditioning that physically enforces social ranking, which precedes ideological awareness. In Joothan, Valmiki describes the difficult operation of the carcass disposal technique where the physical coordination required to move dead cattle in the village without pay involves tying the legs and using a thick bamboo pole, a task requiring the synchronised physical effort of four to six people. In addition, the humiliating tasks are imposed upon Dalit children as techniques of the body to subjugate and designate them in a specific social role. He recounts his childhood when his headmaster forced him to sweep the entire school and the playground throughout his school days instead of educating him. The headmaster defended his action as “family occupation” (Valmiki, 1997, p. 5), basically, exploiting a child’s body to perform and rehearse the community’s “habit of servitude” (Connerton, 1989, p. 10). Talking back at the teacher is considered a punishable action, which Valmiki experienced by being made to squat in the rooster position. These punishments function as a disciplinary mechanism that regulates caste-bound behaviour. Dominant caste authority is sustained through the imposition of hereditary labour as a rigorous form of bodily discipline, ensuring that Dalit subjects repeatedly perform prescribed occupations rather than merely internalising them as social norms. The repeated enforcement of such acts transforms caste hierarchy into an embodied social habit rather than a mere ideological belief. Moreover, Valmiki narrates a traumatic event when he is forced to help his uncle skin a dead bullock, where he undergoes a visceral bodily experience of trembling hands.

“That day something broke inside me. I skinned the bullock under Uncle’s guidance. I felt like I was drowning in a swamp. I was being drawn into the very quagmire that I had tried to escape from. The wounds from the torment that I suffered with uncle on that hot afternoon are still fresh on my skin. As the hide was being skinned, the blood inside me was congealing.” (Valmiki, 1997, p. 41).

His memory of painful performances has carved on each nerve of his brain exhibiting how social hierarchy is physically forced into the nervous system.

For Pawar, the central metaphor of her memoir is *aayadan*, the act of weaving bamboo baskets, which is a repetitive bodily habit inherited through generations of Mahar women. These repetitive practices illustrate incorporated memory, transforming ceaseless labour into an inscribed habitus that reproduces conditions of abject submission. She describes her mother whose fingers “frantically, ceaselessly, weaving baskets, winnows, and other stuff, one after another, stricken with suffering” (Pawar, 2008, Chapter 1). This labour is so deeply incorporated that she continued the physical rhythm even while mourning for her dead son, demonstrating Connerton’s argument that habit involves “bodily automatisms” (Connerton, 1989, p. 5) where awareness retreats. She describes how the striving women of her community performed the repetitive toil of carrying heavy bundles on their heads early in the morning, navigating steep hills like those whose palms are thickened due to searing labour and “feet with huge crevices” (Pawar, 2008, Chapter 1). She notices her sister-in-law with “big knee caps, thin bony legs… and cracked feet” (Pawar, 2008, Chapter 1) are the direct effect of the habit of walking with a heavy load on her head on an everyday basis which are physically marked on their body. Pawar specifically connects her mother’s art of weaving to her own act of writing, suggesting both exhibit pain, suffering, and agony. By transforming this repetitive physical labour into narrative form, she suggests that her prose is an inscription of previously incorporated physical history of pain and suffering.

### 5.3 Spatial subordination: the embodied geography of caste stratification

Connerton’s view on “Choreography of authority” (Connerton, 1989, p. 74) involves “disposition of bodies relative to the bodies of others” (Connerton, 1989, p. 73), where the relative positioning of individuals allows observers and participants to deduce the degree of authority claimed or enjoyed. In these autobiographies, spatial stratification serves as an incorporated practice that sediments social hierarchy directly into the body. In Joothan, the school functions as a site of repetitive postural performance where Dalit Children are forced to embody their outsider status through spatial exclusion. In the classroom, Valmiki was forced to “sit away from the others in the class” (Valmiki, 1997, p. 3) and was strictly prohibited from sitting on chairs, benches, or even the common mat. He was not allowed to sit anywhere but near the door, deported on the bare floor. During his intermediate exams, Valmiki was denied access to the laboratory for practicals, a spatial exclusion that ensured his failure. This physical displacement ensured that his presence was literally on the margins of the educational space, sedimenting a sense of exclusion that made even the letters on the blackboard fade and distant. Furthermore, the restriction to fetch water is a prominent instance of spatial incorporation among casteism. Valmiki was forbidden to touch the water pump, since he is a Dalit, the water pump needed a purification before other castes can use it. This restriction is not just a symbolic act but a disciplinary performance where the upper-caste bodies take authority by managing space and physical distance, to transform social hierarchy into a literal spatial order. Regulating who may touch water or occupy particular spaces ensures that bodily conduct conforms to caste norms through choreography of authority and reproducing hierarchy through everyday repetition. Valmiki was forced to stand at a distance and wait for someone to pour water into his cupped hands. This physical stratification of waiting and the ritualised evasion of touch expresses the concept of pollution as a bodily reality. During school rehearsals and annual functions, Valmiki was kept out and forced to stand outside the door as a spectator. He describes this as the “anguish of standing outside the door” (Valmiki, 1997, p. 19), where his physical location at the threshold performed his status as an outsider to the socially legitimate community. Drawing on Roy (2014), the descending scale of contempt demonstrates such habituated routines by conditioning the subordinated body to perform obedience through repetitive acts of waiting.

Valmiki’s interactions with the Kulkarni family in Ambernath reveal how caste hierarchy is maintained through a spatial subordination that functions as an embodied geography of stratification. Even before his identity as a member of a Scheduled Caste was known, the family practiced a rigid “choreography of authority” (Connerton, 1989, p. 74) by keeping separate dishes for SCs and Muslims who visited their home. This material segregation acts “mnemonics of the body, a constant reminder of the order of estates” (Connerton, 1989, p. 87). The moment he disclosed his true identity, the social and physical atmosphere shifted instantly; he observed that the “distance between us had increased” (Valmiki, 1997, p. 113) and “all links had snapped” (Valmiki, 1997, p. 113). This immediate distancing illustrates how social memory serves to “legitimate a present social order” (Connerton, 1989, p. 3) by re-establishing a “socially specific spatial framework” (Connerton, 1989, p. 37) that defines the subordinate body as inherently separate and inferior. The sudden rift confirms that the inertia of caste is sedimented in the body, allowing ancient beliefs regarding purity and pollution to be reasserted with physical force when the hierarchy is cognitively challenged.

*In The Weave of my life* (2008), the spatial incorporation is apparent in hierarchical steps, a physical architecture of the village which reinforced the habit of servitude. Pawar recounts visiting a Brahmin’s house to buy pickles. The house featured crescent-shaped cement steps that resembled a pyramid from below. As a Dalit child, she was forced to stand at the lowest step, a spatial position that she notes would make “anyone standing at the bottom feel really low” (Pawar, 2008, Chapter 2). The proceeding itself was a ritualised practice of social distance. The Brahmin women would not hand the pickle to her directly; instead, she would place it on the second or third step, which is far away from the least step. Even the exchange of money placed on a step would be touched after sprinkling water on it to cleanse them of pollution. Pawar’s husband, Harishchandra, experienced a disheartening experience of spatial hierarchy when visiting a village Talathi’s home. Despite being a government official, he was told to sit and eat on a verandah covered with grime and filth, chappals and shoes, and goat droppings while his own upper-caste peon was permitted to sit inside the house to eat. Unlike other regions where Dalit houses were at the margins, Pawar mentions that in the Konkan, they were often found at the centre of the village. This was not a symbol of inclusion but a “matter of convenience for the upper castes” (Pawar, 2008, Chapter 1) who wanted the community at their service to summon them for labour at any time. Connerton argues that these culturally specific performances provide a mnemonic of the body, where patterns of authority are recalled through physical orientation. These incidents illustrate the difference between recognising a social code and incorporating it. The frequent act of positioning Dalit bodies by stratifying them to sit on the bare floor, forbidden to touch the water pump, and standing at the bottom step creates inferiority, yet unveils societal ingrained bodily habit. Here, the conscious resistance is lacking and accepts the hierarchy as if the rule is confined within the upper community. During the *Shimga* festival which is identified as another name for the spring festival of Holi, celebrated in the Marathi month of Phalgun (mid-February to mid-March), Mahars were forced to cut down trees yet forbidden to touch the sacred palanquin, which shows as “inertia in social structures” (Connerton, 1989, p. 5), where the society is unwilling for the upliftment of Dalit community.

### 5.4 Gestural incorporation: performed submission and status

Groups maintain their social identity and power structures by entrusting the values they wish to conserve to bodily automatisms. These are habitual gestures, postures, and terminal routines that strip communication of ambiguity and physically enact social rank. In *Joothan*, Valmiki details the salaam custom, a wedding ritual of bowing and begging where a Dalit groom is taken to the houses of higher castes to beg for donations and presents. Connerton argues that to kneel or bow in subordination is not just to state a message of submission but to display it through the “visible, present substance of one’s body” (Connerton, 1989, p. 59). The salaam custom is a performative re-enactment that physically forces the groom into a posture of supplication before the community he serves. Moreover, Dalit individuals were expected to touch the feet of those in power as a gesture of respect from a social subordinate. A young boy shows his gratitude by touching Kureishi’s feet multiple times after the officer intervened to get the boy his unpaid wages. Valmiki describes this as a “conspiracy to trap us in the whirlpool of inferiority” (Valmiki, 1997, p. 39) where the physical substance of the body is used to state and display subordination. Authority is produced through a mutual choreography where the Dalit subject’s ritualised submission such as a groom wedded by a distant priest which confirms the observer’s superior status. This interaction transforms caste hierarchy into an embodied spatial order, reproduced daily through the habituated movements of both dominant and subordinate bodies. Resistance also takes a gestural form when Valmiki’s mother performs a radical act of defiance by overturning a basket of joothan at the feet of a higher-caste man who insulted her. This sudden, aggressive physical movement serves as a “ritual revocation” (Connerton, 1989, p. 13) of her habitual role as a passive recipient of scraps.

Valmiki’s ability to initially pass as a Brahmin in Ambernath demonstrates how status is maintained through gestural incorporation and performative recognition rather than biological markers. Because he possessed the social ability and implicit knowledge of presence that the Kulkarnis associated with their own rank, he was welcomed with “unstinting affection” (Valmiki, 1997, p. 109) most notably during a ritual Diwali bath. In this instance, Mrs. Kulkarni’s act of massaging him with fragrant herbal paste serves as a profound incorporating practice, where a message of inclusion is imparted through “current bodily activity” (Connerton, 1989, p. 72) that exists only as long as the bodies are present to sustain the performance. However, once his identity was revealed, the family’s internalised understanding of caste was totally shocked as they could not no longer reconcile his physical presence with their shared beliefs of the established social order. This transition reveals that while bodily habits can temporarily mask social rifts, they ultimately function to preserve the historical deposit of hierarchy through the habitual memory of the dominant group.

Similarly, Pawar describes a habitual communal tendency to “shrink within ourselves like a tortoise and proceed at a snail’s pace” (Pawar, 2008, Chapter 1) as a bodily response to perpetual insecurity and the threat of attack from upper castes. This postural habit sedimented a sense of vulnerability into the community’s collective bodily deportment. Pawar’s mother-in-law initiated her into the manners of life by insisting to address her younger brothers-in-law in the plural. This linguistic gesture, along with her sister Tai’s adoption of Brahminic honorifics for her husband and mother, as *Aaye*, to the standard Marathi *Aai*, for mother and began using plural honorifics *Apan* to address her husband. These are incorporating practices of “passing” (Connerton, 1989, p. 36), where the individual attempts to overwrite the habit of servitude with the “prestigious ease” (Connerton, 1989, p. 90) of a dominant social code, illustrating how outward proprieties of language are used to incorporate and perform patriarchal and caste-inflected hierarchies. During traditional wedding games, the groom was taught to strike the ground with a stick and demand an explanation for his bride’s lateness. This rehearsed gestural performance functioned as a technique of the body intended to establish and maintain the husband’s authority from the very first day of marriage. Caste and patriarchy regulate Dalit women’s bodies as gateways of the caste system, using ritual expectations and the strict management of sexuality to stabilise the social order. This choreography of authority is enacted through repetitive labour and gestures of obedience that transform the female body into an incorporated site for the daily reproduction of hierarchy. Furthermore, she details the repetitive gesture of taking the dirt from a left toe and pressing it onto a baby’s forehead to avert the “evil eye” (Pawar, 2008, Chapter 6). This “bodily substrate” (Connerton, 1989, p.71) of performance ensured that superstitious categories were kept in mind and passed pre-reflectively to the next generation. These gestural incorporations show that caste is not just an ideology but a physical performance. Whether through the ritualised begging of the salaam, touch of the feet, or linguistic shifts, social memory is sustained by how the body is moved, positioned, and addressed. Thus, authority is reproduced not merely through explicit coercion but through the continuous regulation of everyday bodily performances.

## 6 Habit memory and unconscious transmission

Connerton’s theory of habit memory argues that social memory is something which is carried and preserved through repeated bodily actions and performances rather than an idea. It is “conveyed and sustained by (more or less ritual) performances” (Connerton, 1989, p. 38), which are deeply rooted in the body and transmitted unconsciously. In Dalit narratives, it appears visibly that the social hierarchy is unconsciously embedded in the bodies of communities by performing rituals and everyday habits; the inequity is maintained. According to Connerton, the social memory sustains through “acts of transfer” (p. 39) when rituals are transmitted across generations. In *Joothan*, Valmiki demonstrates his physical pain when his headmaster forced him to sweep the whole school and humiliated as to perform his family occupation. This act reveals the ingrained habit of servitude that had been performed by the community for a long time.

In *The Weave of My Life* (2008), Urmila Pawar describes inheriting the weave of bamboo basket making from her mother, a physical habit that defined their social and economic existence for generations. Meanwhile, memory is transmitted through incorporating practices which are acquired in a way that does “not require explicit reflection on their performance” (Connerton, 1989, p. 102). Dalit children often incorporate their social status through the material milieu of their childhood. Pawar notes that they were aware of their specific caste-bound poverty without anybody telling them, demonstrating that they absorbed their social position through the physical environment and routines of their lives. These mnemonics of the body ensure that patterns of authority are recalled through physical orientation, such as Valmiki being forced to sit on the bare floor near the school door while others sat on mats, a postural habit that sedimented his status as an outsider. Hierarchy is internalised without ideological awareness, as Connerton posits that bodily discipline precedes that, as groups entrust their most important values through bodily automatisms. The choreography of authority is the relative disposition of bodies to communicate rank, stripping the social code of ambiguity. Valmiki’s experience at the school water pump, where he had to wait for someone to pour water into his cupped hands from a distance, enacted the concept of pollution as a physical reality before he could cognitively challenge the religious justifications for it. Even the ritualised consumption of food functions as a mnemonic rehearsal; Valmiki describes how the elders narrated stories of collecting *joothan*, effectively incorporating the habit of servitude among children through their dietary practices. Caste is remembered even when not consciously believed because habit memory functions through bodily automatisms where awareness retreats; caste identities become involuntary and visceral.

## 7 Autobiography as inscription of incorporated memory

Connerton considers inscribing practices such as writing, photography, and print, which are modern devices that “trap and hold information” (Connerton, 1989, p. 73) long after the human organism has stopped informing. In Dalit life writing, the process of inscription is not just a fictional past; rather, it serves the recording of real-time sufferings of caste. Dalit autobiographies act as an intersection of memory where the incorporated practices turn to permanent inscriptive form. As Connerton stated, written words transmit habit memory in the form of knowledge that is “in the hands and in the body” (Connerton, 1989, p. 95), which often escapes conscious notice. Valmiki’s physical trauma of being forced to be in the rooster position as a punishment carved in his brain nerves that exposes the body memory to inscribed one. Meanwhile, Pawar’s description of village women with bulged palms and cracked feet due to heavy work shows that the body’s toil converts to the sufferings of writings. By documenting the torment embedded in the bodily mnemonics of caste, immediate physical suffering is transformed into enduring inscribed records. The shift from oral literature to literate culture in Dalit records identifies the reliability of embodied memories that are preserved in inscribed form. This performs this conversion by taking the traceless social memory of gestures and postures and fixing them in a standard, communicable form. Valmiki takes the choreography of authority, such as being forced to sit on the bare floor while others sit on mats and transforms this postural performance into a narrative testimony that challenges the flat denial in Indian society. Pawar explicitly states that her mother’s act of weaving and her act of writing are linked naturally, which transforms the incorporated physical labour to inscribed form of writing.

These texts perform as a “documented Dalit history” (Valmiki, 1997, p. XLV) and an “archive of Dalit history” (Valmiki, 1997, p. XLVI) that challenges the organised erasure of history in dominant cultural accounts. Valmiki’s archives of consuming *joothan,* collecting, drying, and boiling leftovers functions as a mnemonic of poverty. Similarly, Pawar’s narrative archives of food practices and scarcity, which were performed through gestural fights for one morsel, record the everyday habitual practices within families. Connerton argues that embodied memory is recorded in the form of inscription; it often neglects the memory which is carried by the body. While Dalit autobiographies bridge this gap by documenting real names and incidents, it preserves their testimony of lived suffering. These texts serve as a social document that acts as a depository of larger social phenomena. It ensures that embodied experiences of past generations are preserved rather than forgotten.

## 8 Conclusion

This study concludes by connecting Paul Connerton’s theory of habit memory with Dalit autobiographies of Omprakash Valmiki and Urmila Pawar that casteism is not just an ideology but an embodied mnemonic structure of memory maintained through everyday practices and rituals. Reproducing caste through the concept of habit memory helps to explain the continuity of casteism even after being legally abolished. The primary finding of this analysis is inequality of casteism which is deeply ingrained in the body and performed through repeated gestures, postures, and everyday rituals that do not require conscious thought. Valmiki’s experience of being forced to sweep and Pawar’s description of weaving illustrate the habit of servitude becoming physically internalised and passed down across generations. Furthermore, this research highlights the “choreography of authority” (Connerton, 1989, p. 74), where the positioning of bodies in space determines the social hierarchy that is visible in ranking the community. This is evident in the ritualised distance at the water pump in *Joothan* and the lowest step of the Brahmin’s house in *The Weave of My Life*. These are legitimating performances where the body serves as a substrate for status, stripping social codes of ambiguity. Because these habits are acquired pre-reflectively, caste is remembered viscerally, as seen in Valmiki’s adult reaction of nausea when recalling the food of upper castes. This study contributes to the sociology of stratification by demonstrating the limited role of inscribing practices. While religious texts and laws describe caste and abolishment, it continues to persist because of the reproduction through everyday bodily practices. Thorat and Attewell (2007) find Dalit applicants face systematic hiring discrimination even with equal qualifications through their audit which demonstrates that caste hierarchy is reproduced through hidden, social attitudes and institutional practices. Connerton’s (1989) concept of “inertia in social structures” (p. 5) explains this existence when the repeated bodily habits pile up as a historical layer in the body that resists change. Here, Dalit life writings play a vital role by transforming invisible, embodied memory to written narratives which make them permanent records. Through their writing, Valmiki and Pawar effectively convey the forgotten memory of the body and exhibit it as sociological evidence which challenges the organised erasure of dominant history. In modern society, even when discrimination is recognised, it is considered leftover problem of the past which leads to neglecting the fact that economically backward Dalits still lack in education and legal safeguards due to poverty which showcases discrimination persists beyond laws. Future research can explore the hysteresis effect, where old temperaments sustain even when social conditions change, especially in digital contexts and modern urban areas. Furthermore, scholars can study how new bodily practices, such as adapting to a new professional environment or to elite status, interact or conflict with inherited habit memories as Pawar rightly stated, caste may appear absent in cities but debris sustains in hidden ways and ready to re-emerge. Additionally, 1956 Buddhist conversion may help scholars to examine how communities consciously reshape their identity from deeply rooted bodily habits.

## Data Availability

Publicly available datasets were analysed in this study. This data can be found at: Valmiki (1997) and Pawar (2008).
